# On the Discovery of TOR As the Target of Rapamycin

**DOI:** 10.1371/journal.ppat.1005245

**Published:** 2015-11-05

**Authors:** Joseph Heitman

**Affiliations:** 1 Departments of Molecular Genetics and Microbiology (MGM), Pharmacology and Cancer Biology, and Medicine, Duke University Medical Center, Durham, North Carolina, United States of America; 2 Tri-Institutional (Duke, UNC, NC State) Molecular Mycology and Pathogenesis Training Program (Tri-I MMPTP), Duke University Medical Center, Durham, North Carolina, United States of America

Louis Pasteur, who discovered microbes cause disease and developed early vaccines, said, “Chance favors the prepared mind.” With this in mind, I joined the physician-scientist MD-PhD training program at Cornell and Rockefeller Universities in 1984. The National Institutes of Health (NIH) launched the medical scientist training program in 1964 to train physician-scientists as ambassadors between the disparate worlds of science and medicine. But in my case, after completing the first half of medical school and graduate school in 1989, something seemed to be missing. I took a leave of absence from medical school to conduct scientific studies at the Biocenter, University of Basel, Switzerland. This sojourn led to the discovery of mechanisms of action and targets for a new class of drugs used in patients who receive organ transplants, stents for heart disease, and cancer chemotherapy.

My research addressed a simple question: how do cells sense their environment and transfer information inside the cell? Our focus was also simple: the yeast *Saccharomyces cerevisiae* (baker’s yeast). Although this is an outstanding model, our studies failed to produce the insights we sought. As the future in science began to appear bleak, I contemplated returning to medical school early.

But before conceding failure, I began considering an alternate approach. Could we use some chemicals, or drugs, to understand how cells transfer information? While reading in the library, I came upon a new article in the journal *Nature* showing the drug cyclosporin could be studied in a similar fungus. I knew from medical school that cyclosporin was a drug given to patients to prevent rejection of transplanted organs, and that it acted on a specific cell of our immune system, but how it did so was a mystery. I returned to the lab, saying that we had to work on this!

I was in the right place at the right time. My advisor, Mike Hall, had been hired to consult for Sandoz Pharmaceuticals, also located in Basel. Scientists at this company discovered and developed cyclosporin as the gold-standard drug for organ transplant patients. Rao Movva was a scientist at Sandoz who had begun studying if yeast could be used to understand how this drug works. He and I met, discussed the projects, and immediately began collaborating to identify targets of immunosuppressive drugs, including cyclosporin and two experimental immunosuppressants, FK506 and rapamycin, not yet used in patients.

All three immunosuppressants are natural products of microbes that live in the soil. All three have potent antifungal activity. Our hypothesis was that they evolved to inhibit growth of competing microbes in the soil, not to do harm to animals. We envisioned harnessing the power of yeast genetics to isolate mutants resistant to each drug and to thereby identify their targets and mechanisms of action. We hypothesized that these drugs inhibit the same proteins in yeast cells as in our immune cells, and that by this approach we could understand how these drugs work. And we envisioned that this information would stimulate further medical advances and new treatments for human disease.

Our approach succeeded spectacularly. With rapamycin, we isolated a large collection of resistant yeast mutants. Through their analysis, we identified three target proteins. The first is an abundant small protein, FKBP12, that serves as a cellular receptor for the drug, forming an FKBP12-rapamycin complex. FKBP12 is conserved from yeast to humans. Yet because it is abundant and present in all cells in the human body, at the time, many immunologists thought it unlikely to be involved in specific drug action. Our yeast genetic studies showed that mutants lacking FKBP12 were viable and completely drug resistant, proving that FKBP12 is essential for drug action.

Our studies revealed two other novel proteins, named TOR1 and TOR2 for target of rapamycin. TOR also means door or gateway in German, and the TOR protein serves as a gateway to cell growth and proliferation. This name also commemorates the city in which TOR was discovered, as Basel is an older European city once ringed by a protective wall with large decorative gates, including one still standing, named the Spalentor. Our genetic and later biochemical studies demonstrated that TOR is the direct target of the FKBP12-rapamycin complex. Several years later, five groups converged to identify the related protein from mammals, including humans, now known as the mammalian target of rapamycin (mTOR). Our subsequent studies (with Maria Cardenas at Duke), and those of others, showed that TOR is a protein kinase localized on intracellular membranes that senses nutrients (amino acids) and governs appropriate physiological responses in cells and animals. Inhibition of TOR by FKBP12-rapamycin blocks fungal growth and suppresses immune responses.

Following these studies, FK506 was FDA approved in 1994 and rapamycin in 1999 for organ transplant recipients to prevent rejection. Rapamycin and its analogs have additional indications in interventional cardiology to prevent coronary artery restenosis and as cancer chemotherapy drugs. Rapamycin inhibits nutrient sensing by TOR, mimicking caloric restriction. Mice fed rapamycin live longer, suggesting aging might someday be reversed with drugs.

As this story illustrates, it is difficult to predict where breakthroughs in medicine will come from, but one certainty is investments in education, training, and basic-science–driven discovery often reap dividends far beyond the original cost, by enabling chance to favor prepared minds.

**Image 1:**
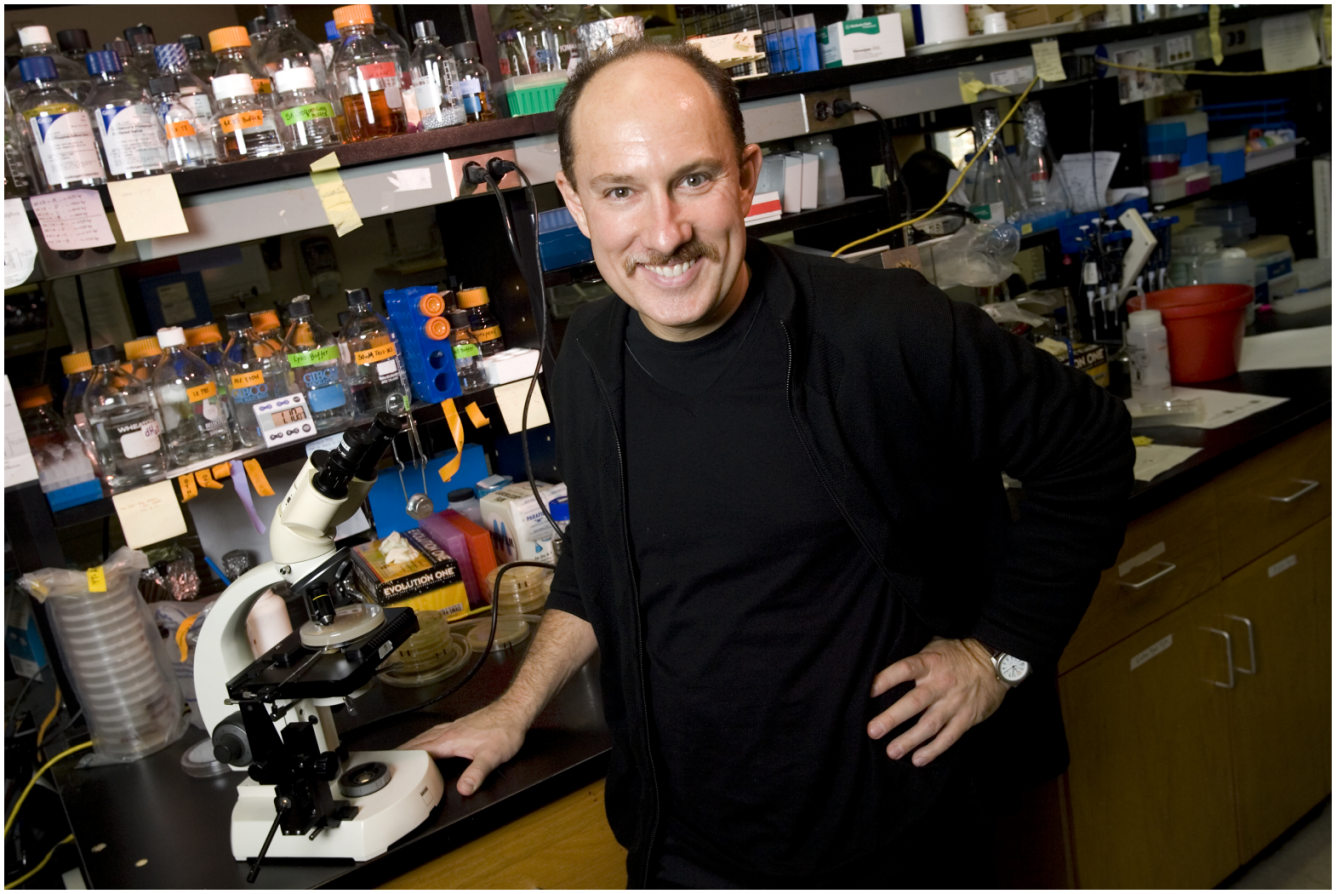
Joseph Heitman.

